# Benefits and Risks of Rapid Initiation of Antiretroviral Therapy: A Systematic Review and Meta-Analysis

**DOI:** 10.3389/fphar.2022.898449

**Published:** 2022-06-03

**Authors:** Ruojing Bai, Juan Du, Shiyun Lv, Wei Hua, Lili Dai, Hao Wu

**Affiliations:** ^1^ Beijing Key Laboratory for HIV/AIDS Research, Center for Infectious Diseases, Beijing Youan Hospital, Capital Medical University, Beijing, China; ^2^ Travel Clinic, Center for Infectious Diseases, Beijing Youan Hospital, Capital Medical University, Beijing, China

**Keywords:** HIV, rapid, antiretroviral therapy, systematic review, meta-analysis

## Abstract

**Objectives:** To compare the benefits and risks between Rapid ART and standard/delayed treatment for HIV.

**Methods:** Databases of PubMed, Cochrane Library, Embase and Web of science were searched from the inception to 28 October 2021. Two investigators independently screened studies related to Rapid ART, extracted data, and evaluated the literature quality. The risk of bias was assessed by Cochrane Collaboration Risk of Bias Tool and the statistical software Stata15.0 was used for meta-analysis.

**Results:** Ten eligible studies were included in this meta-analysis, the results showed Rapid ART was superior to standard/delayed treatment in continuing care for at least 8 months (RR = 1.13, 95%CI: 1.03∼1.25, Z = 2.44, *p* = 0.015), and severe bacterial infection (RR = 0.42, 95%CI: 0.25∼0.70, Z = 3.33, *p* = 0.001). At 12 months following treatment, there was no statistically significant difference in viral load <100 copies/mL (RR = 1.05, 95%CI: 0.80∼1.39, Z = 0.35, *p* = 0.726), mortality (RR = 0.77, 95%CI: 0.47∼1.24, Z = 1.09, *p* = 0.277), or the incidence of adverse events (RR = 0.52, 95%CI: 0.16∼1.76, Z = 1.05, *p* = 0.294) compared with standard/delayed treatment.

**Conclusion:** In comparison to standard/delayed treatment, rapid ART can reduce the incidence of TB and severe bacterial infections in HIV patients. Our findings suggest that rapid ART should be utilized when clinical conditions and the patient’s physical state allow.

**Systematic Review Registration:** [https://inplasy.com/?s=202210004], identifier [INPLASY202210004].

## Introduction

The World Health Organization (WHO) estimates that 37.7 million people globally will be diagnosed with HIV by 2020. Antiretroviral therapy (ART) has been shown to significantly decrease the mortality and transmission of HIV. By the end of 2020, 73% (56–88%) of HIV-infected people were receiving antiretroviral therapy, up from 25% (18–30%) in 2010. However, there were still 1.5 million HIV infections and approximately 68,000 deaths worldwide in 2020 (WHO, 2021).

In 2015, based on the START and TEMPRANO trial data, WHO recommended rapid initiation of ART regardless of CD4^+^ T cell count, that is, starting treatment within 7 days after diagnosis and encouraging the initiation on the same day (Wilkinson et al., 2015). Currently, there is no uniform and clear definition of the time of Rapid ART. Immediate and same-day ART should refer to within 24 h after diagnosis, but due to practical reasons, such as the complexity of providing health care services or formulating diagnostic examination protocols, the actual initiation time may be 5, 7, 14, 28 days or longer after diagnosis. For example, the START protocol stipulates that the rapid initiation group should start treatment within 60 days (Lundgren et al., 2015).

The CD4^+^ T lymphocytes of normal adults are 500∼1,600/µL, while in HIV-infected patients, there is a progressive or irregular decline in CD4^+^ T lymphocytes. In most international guidelines, the threshold of CD4^+^ T lymphocytes for initiating ART was defined as 350/mm^3^ for the years 2006–2009 and increased to 500/mm^3^ in 2009–2013. An important step was taken between 2012 and 2015 to recommend treatment regardless of the CD4^+^ T lymphocyte count (Eholié et al., 2016). As the result, several international trials defined immediate treatment as Rapid ART, standard treatment as CD4^+^ T lymphocyte threshold of 500/mm^3^, and delayed treatment as CD4^+^ T lymphocyte threshold of 350/mm (Lundgren et al., 2015; Borges et al., 2016; Eholié et al., 2016; Boatman et al., 2019; Amstutz et al., 2020; Maskew et al., 2020).

Continuing care in AIDS treatment is an ongoing challenge to global public health (Rosen and Fox, 2011), and is the key to ensuring the success of ART. Currently, there is still controversy over the care retention rate of Rapid ART. A systematic review based on randomized controlled trials showed that Rapid ART improved care retention for 12 months (Mateo-Urdiales et al., 2019), while an observational study in southern Africa found a lower care retention rate (Joseph Davey et al., 2020), which may be related to the living standards.

HIV viral load measures the amount of virus in the blood, used to monitor the level of virus replication and the effectiveness of ART. The goal of treatment is to reduce the viral load in the blood to an undetectable level (less than 50 copies/mL) (Mateo-Urdiales et al., 2019). However, the thresholds used to define virus inhibition in different settings and periods are inconsistent. The CASCADE HIV trial defined viral inhibition as viral load <100 copies/mL (Labhardt et al., 2018; Amstutz et al., 2020), while in several HIV trials in the United States, viral inhibition was defined as viral load <200 copies/mL (Pilcher et al., 2017; Bacon et al., 2020; McNulty et al., 2020).

HIV weakens a person’s immunity to opportunistic infections, such as tuberculosis and fungal infections, severe bacterial infections, and certain cancers. The leading causes of death among adults with advanced HIV globally include tuberculosis (TB), severe bacterial infection, cryptococcal meningitis, toxoplasmosis, and pneumocystis carinii pneumonia (PCP) (Ford et al., 2015; Low et al., 2016). Several systematic reviews and meta-analyses utilized mortality and continuing care as outcome measures, but the conclusions are controversial (Ford et al., 2018; Mateo-Urdiales et al., 2019; Long et al., 2020). On this basis, mortality, continuing care, adverse events, viral load <100 copies/mL at 12 months following treatment, tuberculosis, and severe bacterial infections were included as outcome measures in this meta-analysis.

Rapid ART has been recommended for the treatment of HIV, but its efficacy and safety remain controversial. Therefore, we conducted this systematic review and meta-analysis to assess the advantages and disadvantages of Rapid ART against standard/delayed treatment and to offer a reliable reference for active treatment decisions.

## Methods

### Meta Registration

This meta-analysis was conducted in accordance with the PRISMA (Preferred Reporting Items for Systematic Reviews and Meta-Analyses) statement. The protocol for the study has been submitted to INPLASY PROTOCOL (registration number INPLASY202210004).

### Research Objective

The English databases were searched for publicly published studies comparing Rapid ART and standard/delayed treatment in HIV patients, with grey literature excluded.

### Literature Inclusion and Exclusion Criteria

Literature inclusion criteria: 1) Rapid ART was administered to the intervention group (immediate/same-day diagnosis and treatment), while the control group was treated with standard/delayed treatment; 2) Articles in English only; 3) No restrictions on gender, age and region.

Literature exclusion criteria: 1) Non-English literature; 2) Duplicate, irrelevant literature and unavailable original literature; 3) Interventions to prevent mother-to-child transmission and improve the initiation of antiretroviral therapy in pregnant women; 4) Individuals receiving antiretroviral therapy for reasons other than the treatment of HIV infection (e.g., ART for post-exposure prophylaxis and pre-exposure prophylaxis), and studies including patients with co-infection who were not recommended to receive rapid ART treatment for clinical reasons (e.g., cryptococcal meningitis); 5) The minimum sample size was defined as at least 20 patients.

### Literature Retrieval

The literature was collected from English databases such as PubMed, Cochrane Library, Embase, and Web of Science, with the search period set to 28 October 2021. The retrieval strategy used was subject terms + free words, and the subject keywords in PubMed were HIV: “HIV” [Mesh], and anti-retrovirus: “Anti-Retroviral Agents” (Mesh), and the free words without subject terms were “immediate” (Title/Abstract) and “same-day” (Title/Abstract).

### Literature Screening and Data Extraction

In our investigation, the entire process of literature screening was conducted by two independent researchers. The first stage of screening was conducted according to the title and abstract to eliminate non-randomized controlled trials, and studies with inconsistent experimental interventions, lack of controls, or failing to match the theme. Then, the included articles were screened according to the full text, and finally the included literature of this study was selected.

Using standardized forms, two researchers independently retrieved data from the included articles. The retrieved data primarily consisted of: basic study information (first author, publication year, country, etc.), patient information (number of patients, gender ratio, mean age), interventions, and outcome measures. The two researchers cross-checked after completing the information extraction, and any disagreement was adjudicated by a third researcher Supplementary Data Sheet 1.

### Outcome Measures

Viral load <100 copies/mL at 12 months following treatment, continuing care, mortality, adverse events, infected tuberculosis, and severe bacterial infections were all the outcome measures of this meta-analysis.

### Quality Evaluation

The two researchers independently used the Cochrane Collaboration Risk of Bias Tool (CCRBT) (Cumpston et al., 2019) to assess the risk of bias in the included articles. Following the completion of the evaluation, the two researchers cross-checked their findings, and if there was any controversy, a third researcher participated in the decision-making process. The Cochrane Collaboration Risk of Bias Tool examined the risk of bias specifically using seven items designed from the six aspects listed below: 1) Selection bias (Random sequence generation, Allocation concealment), 2) Performance bias (Blinding of participants and personnel), 3) Detection bias (Blinding of outcome assessment), 4) Attrition bias (Incomplete outcome data), 5) Reporting bias (Selective reporting), 6) Other bias. The risk of bias assessment resulted in “high risk,” “low risk,” and “unclear” for each item.

### Statistical Methods

Meta-analysis was performed on the data with Stata15.0 software. In this Meta-analysis, the combined effect sizes of viral load <100 copies/mL, continuing care, mortality, adverse events, infected tuberculosis, severe bacterial infections were expressed by the relative risk rate (RR) and its 95% confidence interval, namely RR (95% CI).

*Q* test and *I* ^*2*^ were used to quantify the heterogeneity among different studies. When *I* ^*2*^ < 50% or *p* > 0.05, a fixed-effect model was used to combine the outcome measures; and when *I* ^*2*^ ≥ 50% or *p* < 0.05, a random-effect model was adopted to combine the outcome measures.

## Results

### Literature Retrieval Results

**FIGURE 1:**
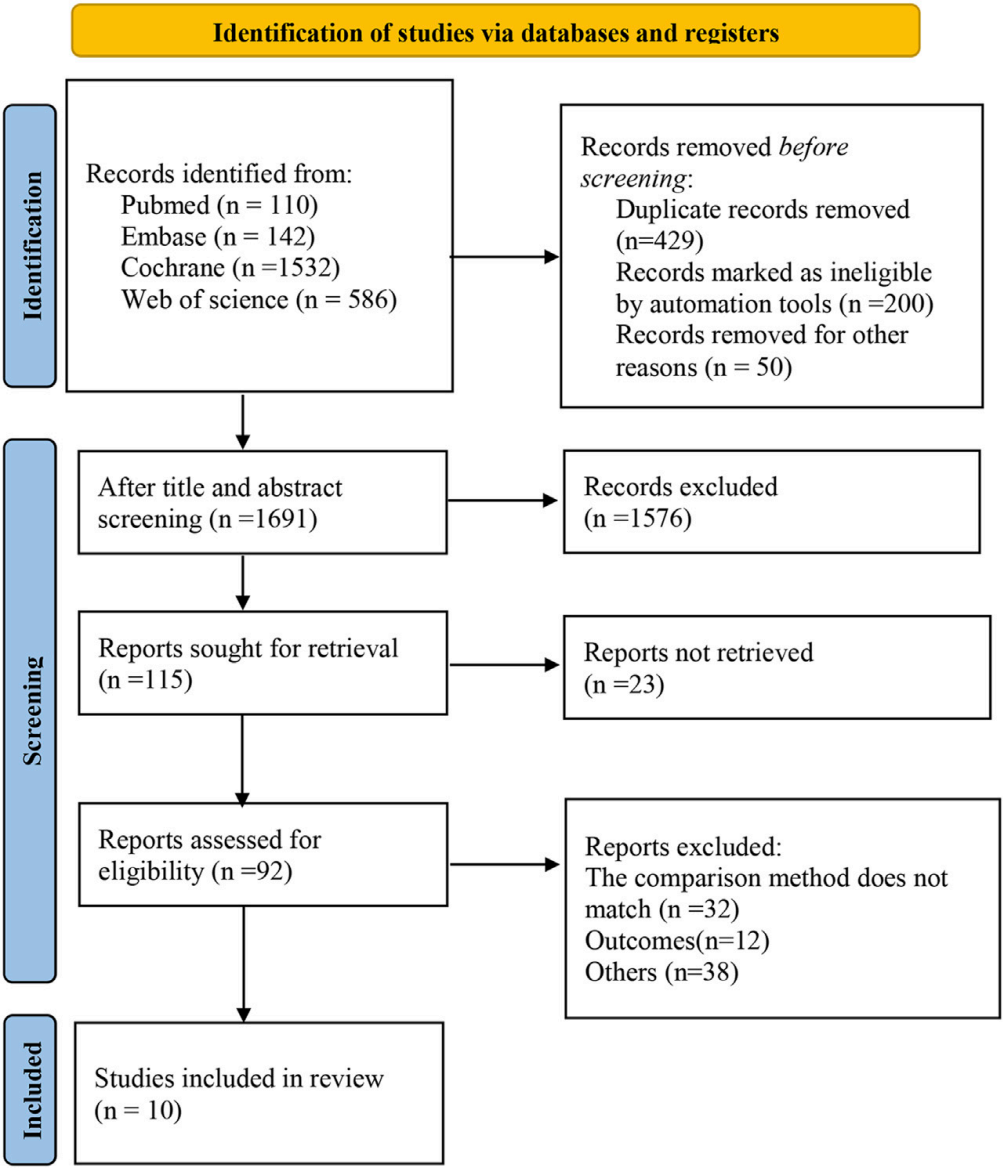
Literature screening process and results.

The databases yielded a total of 2,370 relevant studies, and 10 studies (Boatman et al., 2019; Amstutz et al., 2020; Maskew et al., 2020; Borges et al., 2016; Labhardt et al., 2018; The TEMPRANO ANRS 12136 Study Group, 2015; Koenig et al., 2017; Hecht et al., 2006; Lifson et al., 2017; O’Connor et al., 2017) were finally included for this meta-analysis after the gradual screening. Figure 1 depicts the literature screening process.

### Basic Characteristics of Included Literature

A total of 21,543 patients were enrolled in the included studies from 2006 to 2020, including 10,684 in the experimental group and 10,859 in the control group. Five studies (Amstutz et al., 2020; Maskew et al., 2020; Labhardt et al., 2018; The TEMPRANO ANRS 12136 Study Group, 2015; Koenig et al., 2017) involved patients from resource-limited African regions (Haiti, South Africa, Lesotho, Ivory Coast), and five multi-center studies (Hecht et al., 2006; Borges et al., 2016; Lifson et al., 2017; O’Connor et al., 2017; Boatman et al., 2019; Amstutz et al., 2020; Maskew et al., 2020) involved patients from multiple regions. Nine randomized controlled trials (Boatman et al., 2019; Amstutz et al., 2020; Maskew et al., 2020; Borges et al., 2016; Labhardt et al., 2018; The TEMPRANO ANRS 12136 Study Group, 2015; Koenig et al., 2017; Lifson et al., 2017; O’Connor et al., 2017) were included, of which three (Labhardt et al., 2018; The TEMPRANO ANRS 12136 Study Group, 2015; Koenig et al., 2017) were non-blind, with rapid initiation of antiretroviral therapy in the intervention group and standard/delayed treatment in the control group. All of the treatments lasted longer than 6 months (see Table 1).

**TABLE 1:** Basic characteristics of included literature.

No.	Author	Year	Author country	Patient country	Design	Intervention		N		Sex(man/female)		Age-Median (IQR)		Follow up time
						I	C	I	C	I	C	I	C	
1	Jemma O’Connor	2017	United Kingdom	Asia, Australia, Europe and Israel, North America, South America	RCT	Immediate ART	Deferred ART	2,326	2,359	3,428/1,257		36 (29–44)		median 2.8 years
2	Serena P. Koenig	2017	Haiti	Haitian	Unblinded, RCT	Same-day ART initiation	Standard ART initiation	347	356	181/166	175/181	37 (29, 46)	37 (30, 45)	3.0 years
3	TEMPRANO ANRS 12136 Study Group	2015	Ivory Coast	multicenter, Ivory Coast	Unblinded, RCT	immediate ART initiation	Deferred ART	515	511	108/407	111/400	35(30–42)	35(29–41)	30 months
4	Álvaro H. Borges	2016	Denmark	35 countries	RCT	Immediate cART	Deferred arm	2,326	2,359	3,428/1,257		36 (29–44)		3.0 years
5	Frederick M. Hecht	2006	United States	Multicenter	Cohort study	Acute treatment	Early treatment	13	45	13/0	Jun-39	34(18–56)	34(20–60)	72 weeks
6	Jeffrey A. Boatman	2018	United States	United States, Africa, Asia, Europe and Israel, Australia, Latin America	RCT	Immediate ART group	Deferred initiation	2,325	2,359	—	—	—	—	8 months
7	Mhairi Maskew	2020	South Africa	South Africa	RCT	Intervention arm	Standard arm	296	297	112/184	107/189	35 (29–41)	35 (30–44)	—
8	Alan R. LIFSON	2017	United States	35 countries	RCT	The immediate group	The deferred group	2,262	2,299	1,662/600	1,678/621	36	36	3 years
9	Alain Amstutz	2020	Switzerland	Lesotho	RCT	Same day arm	Usual care arm	137	137	94/180		39 (28–52)		24 months
10	Niklaus D. Labhardt	2018	Switzerland	Lesotho	Open-label RCT	Same-day home-based ART initiation	Usual care	137	137	47/90	47/90	41(31–53)	38 (28–50)	14 months

### Quality Evaluation of Included Literature

**FIGURE 2:**
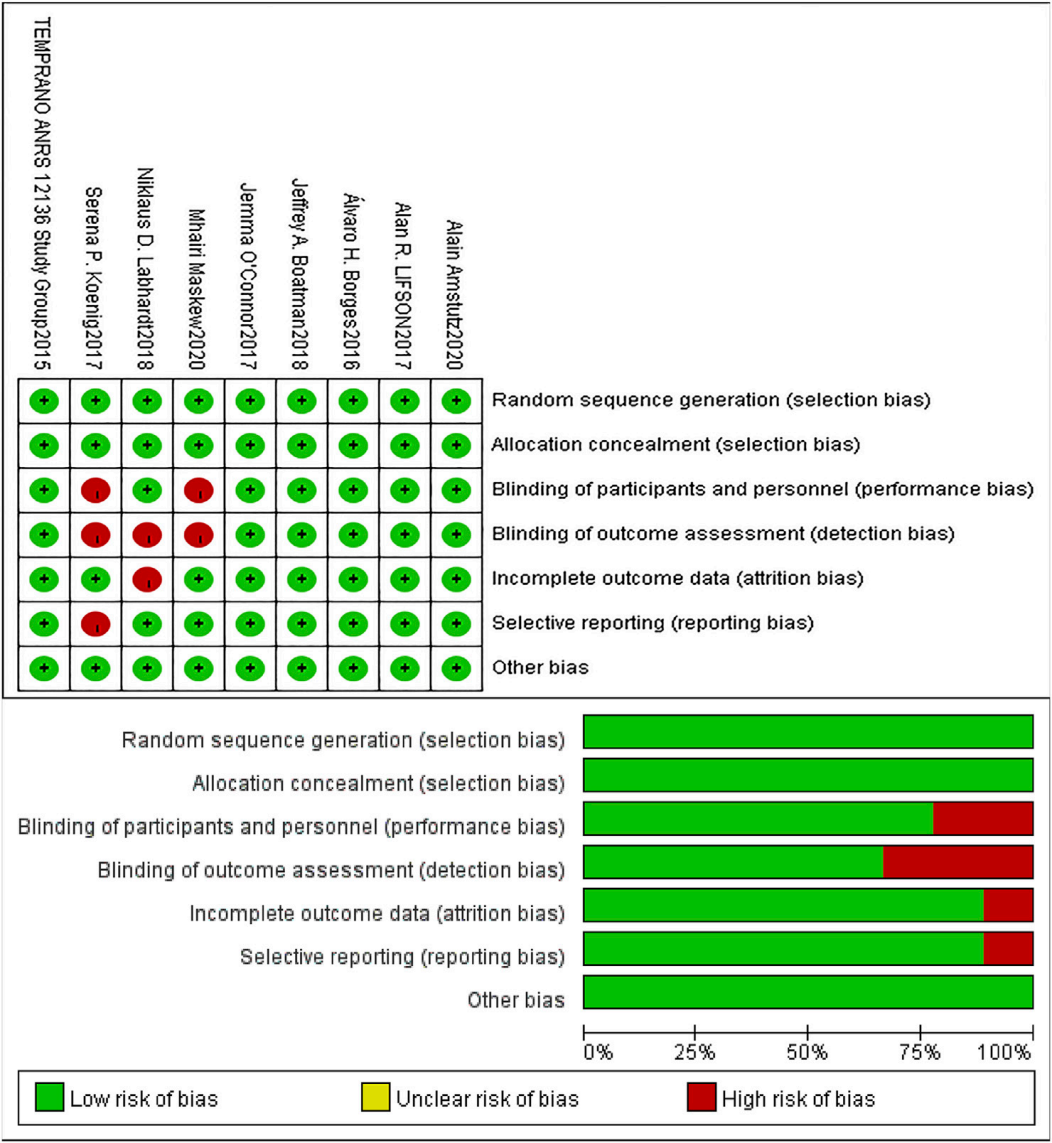
Risk of bias summary and graph.

The risk of bias in the included studies was assessed using the Cochrane Collaboration Risk of Bias Tool (Higgins et al., 2019). Table 2 shows the risk ratings of Selection bias, Performance bias, Detection bias, Attrition bias, Reporting bias and Other bias in each study, and the graphics were displayed by Revman 5.4. (See Table 2; Figure 2).

**TABLE 2:** Assessment of risk of bias in the included literature.

No	Author	Year	V1	V2	V3	V4	V5	V6	V7
1	Jemma O’Connor	2017	LOW	LOW	LOW	LOW	LOW	LOW	LOW
2	Serena P. Koenig	2017	LOW	LOW	HIGH	HIGH	LOW	HIGH	LOW
3	TEMPRANO ANRS 12136 Study Group	2015	LOW	LOW	LOW	LOW	LOW	LOW	LOW
4	Álvaro H. Borges	2016	LOW	LOW	LOW	LOW	LOW	LOW	LOW
6	Jeffrey A. Boatman	2018	LOW	LOW	LOW	LOW	LOW	LOW	LOW
7	Mhairi Maskew	2020	LOW	LOW	HIGH	HIGH	LOW	LOW	LOW
8	Alan R. LIFSON	2017	LOW	LOW	LOW	LOW	LOW	LOW	LOW
9	Alain Amstutz	2020	LOW	LOW	LOW	LOW	LOW	LOW	LOW
10	Niklaus D. Labhardt	2018	LOW	LOW	LOW	HIGH	HIGH	LOW	LOW

Note: V1-V7 in the table represents Random sequence generation, Allocation concealment, Performance Blinding of participants and personnel, Blinding of outcome assessment, Incomplete outcome data, Selective reporting and Other bias in sequence.

### Viral Load <100 Copies/mL at 12 Months After Treatment

**FIGURE 3:**
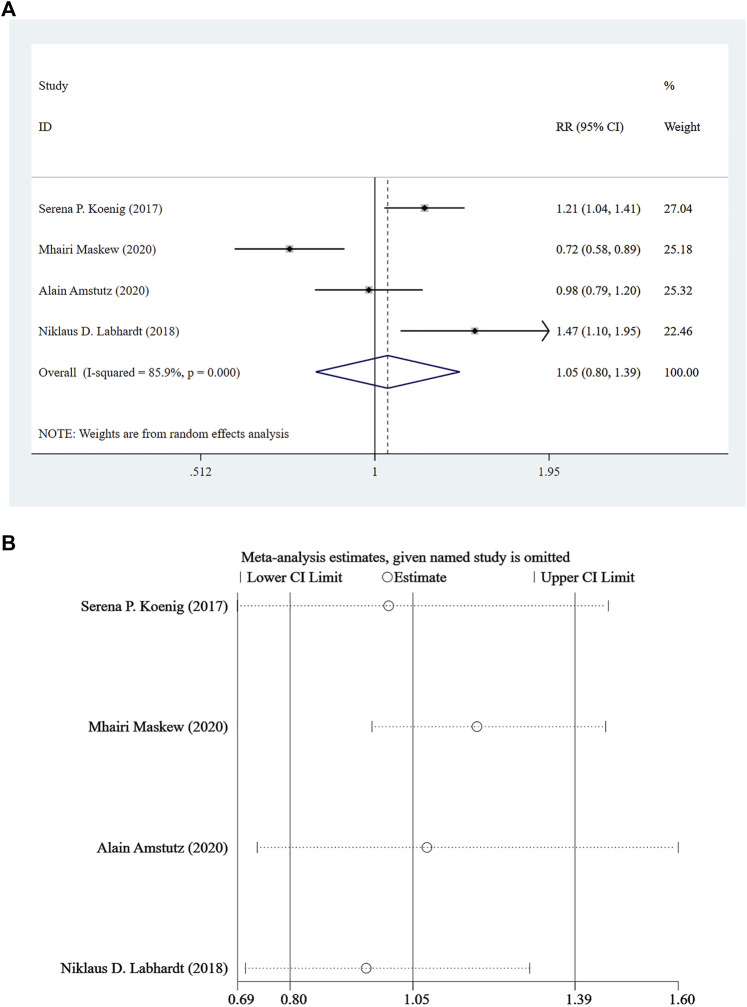
**(A)** Forest plot for Meta-analysis of statistical chart of viral load <100 copies/mL at 12 months after treatment. **(B)** Sensitivity analysis of statistical chart of viral load <100 copies/mL at 12 months after treatment (After removing the literatures one by one, the circles of each study were within the two edges, indicating that the conclusions of this meta-analysis were stable and reliable).

Four studies (Koenig et al., 2017; Labhardt et al., 2018; Amstutz et al., 2020; Maskew et al., 2020) reported viral load <100 copies/mL at 12 months following treatment, and a random-effect model was adopted for the combined effect size (*I* ^*2*^ = 85.9%, *p* < 0.001). There was no significant difference between the two groups based on the combined results (RR = 1.05, 95% CI: 0.80–1.39, Z = 0.35, *p* = 0.726), suggesting that the risk of Rapid ART with viral load <100 copies/mL was close to that of standard/delayed treatment, as shown in Figure 3A and Figure 3B.

### Continuing Care

**FIGURE 4:**
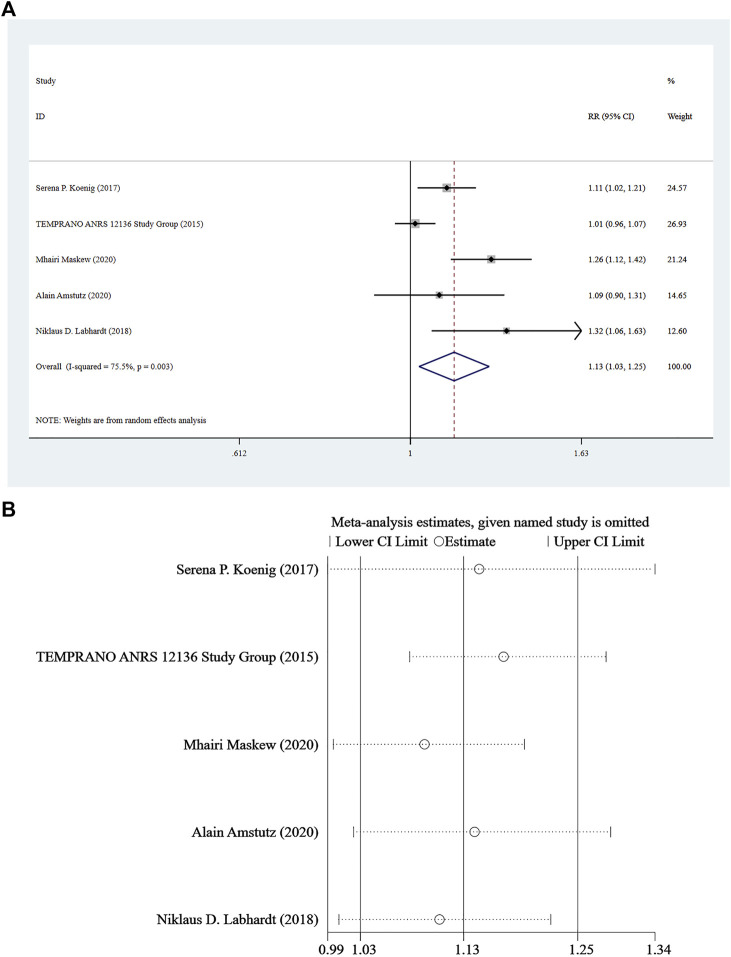
**(A)** Forest plot for Meta-analysis of continuing care statistics chart. **(B)** Sensitivity analysis of continuing care statistics chart (After removing the literatures one by one, the circles of each study were within the two edges, indicating that the conclusions of this meta-analysis were stable and reliable).

Five studies (Amstutz et al., 2020; Maskew et al., 2020; Labhardt et al., 2018; The TEMPRANO ANRS 12136 Study Group, 2015; Koenig et al., 2017) reported continuing care for at least 8 months, and a random-effect model was used for the combined effect size (*I* ^*2*^ = 75.5%, *p* = 0.003). The pooled results showed that the difference between the two groups was statistically significant (RR = 1.13, 95% CI: 1.03–1.25, Z = 2.44, *p* = 0.015), indicating that Rapid ART could be considered superior to standard/delayed treatment in the risk of continuing care. See Figure 4A and Figure 4B.

### Mortality

**FIGURE 5:**
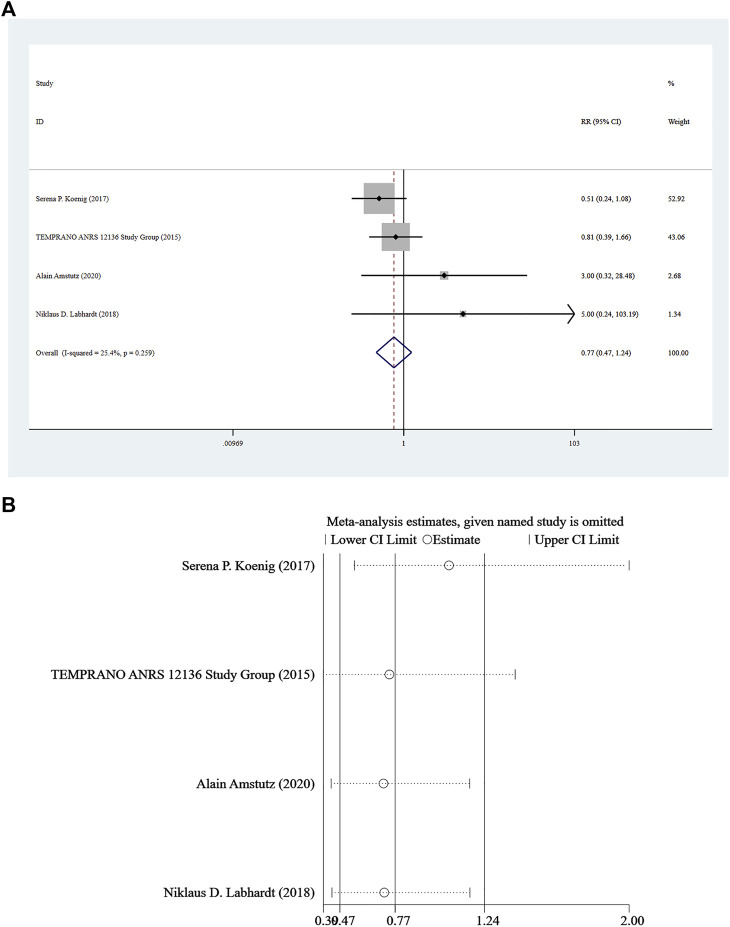
**(A)** Forest plot for Meta-analysis of mortality chart. **(B)** Sensitivity analysis of mortality chart (After removing the literatures one by one, the circles of each study were within the two edges, indicating that the conclusions of this meta-analysis were stable and reliable).

Mortality was reported in four studies (Amstutz et al., 2020; Labhardt et al., 2018; The TEMPRANO ANRS 12136 Study Group, 2015; Koenig et al., 2017), and a fixed-effect model was adopted for the combined effect size (*I* ^*2*^ = 25.4%, *p* = 0.259). There was no significant difference between the two groups based on the combined results (RR = 0.77, 95%CI: 0.47–1.24, Z = 1.09, *p* = 0.277), suggesting that the risk of death associated with Rapid ART was similar to that of standard/delayed treatment. See Figure 5A and Figure 5B.

### Adverse Events

**FIGURE 6:**
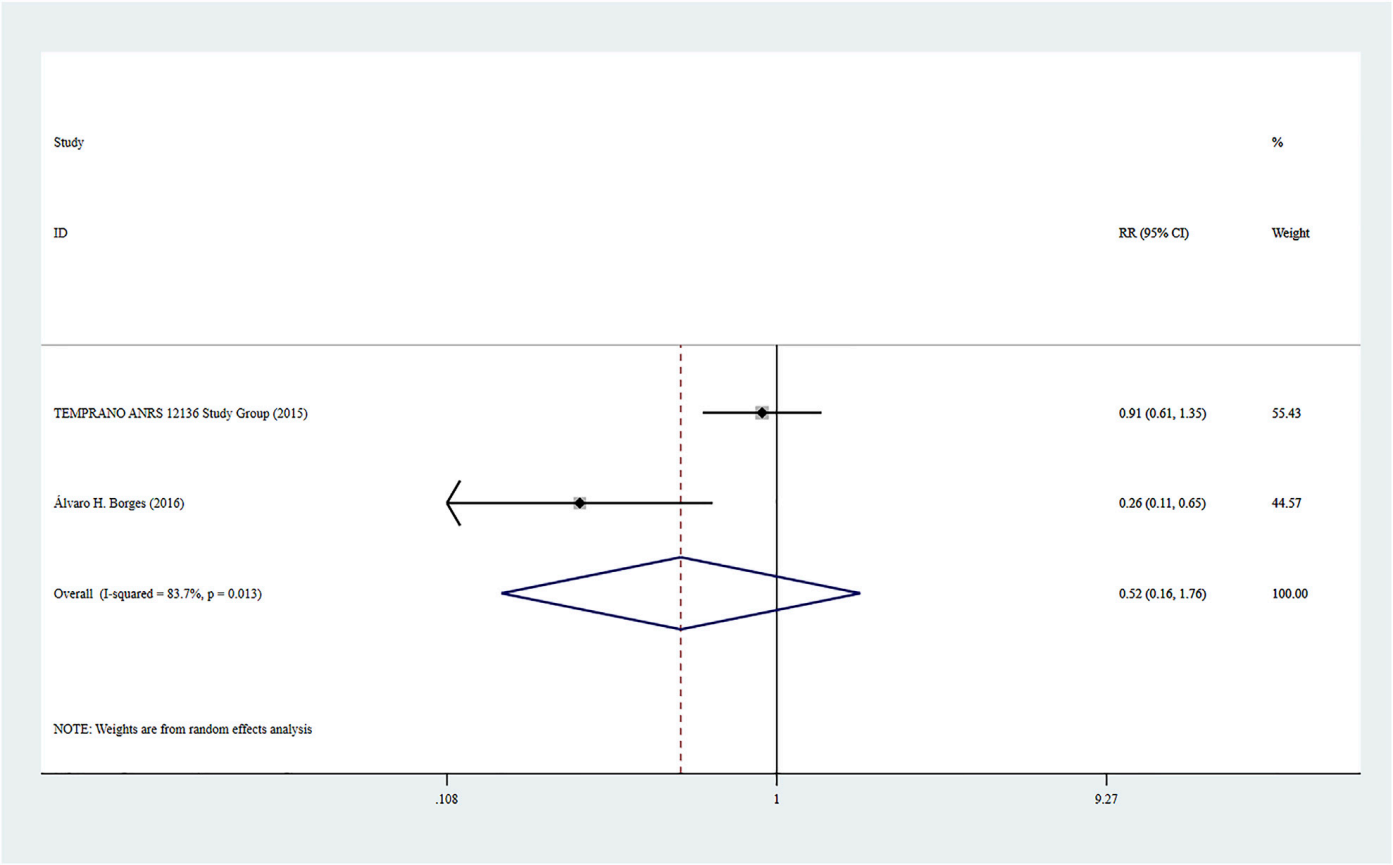
Forest plot for Meta-analysis of adverse events.

Adverse events were reported in two studies (8, 21), and the combined effect size was based on a random-effect model (*I* ^*2*^ = 83.7%, *p* = 0.013). There was no significant difference between the two groups based on the combined results (RR = 0.52, 95% CI: 0.16–1.76, Z = 1.05, *p* = 0.294), suggesting that the risk of adverse events caused by Rapid ART was close to that of standard/delayed treatment. See Figure 6.

### Infected Tuberculosis

**FIGURE 7:**
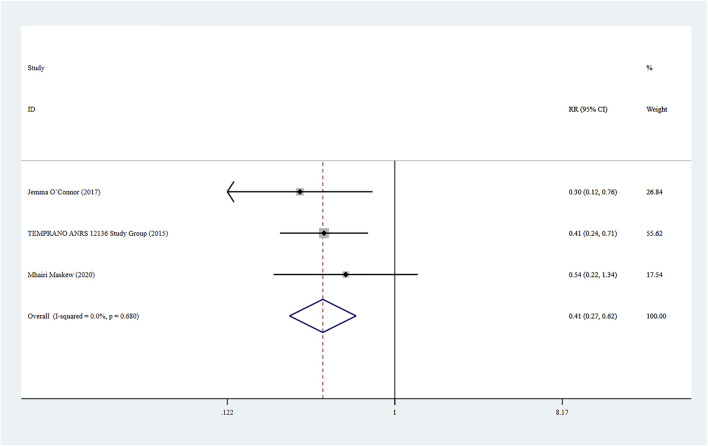
Forest plot for Meta-analysis of infected tuberculosis.

Three studies (Maskew et al., 2020; The TEMPRANO ANRS 12136 Study Group, 2015; O’Connor et al., 2017) reported infected tuberculosis in HIV patients, and a fixed-effect model was adopted for the combined effect size (*I* ^*2*^ < 0.01%, *p* = 0.680). The combined results showed a statistically significant difference between the two groups (RR = 0.41, 95% CI: 0.27–0.62, Z = 4.24, *p* < 0.001), suggesting that the risk of infected tuberculosis caused by Rapid ART group was lower than that of standard/delayed treatment group, as shown in Figure 7.

### Severe Bacterial Infections

**FIGURE 8:**
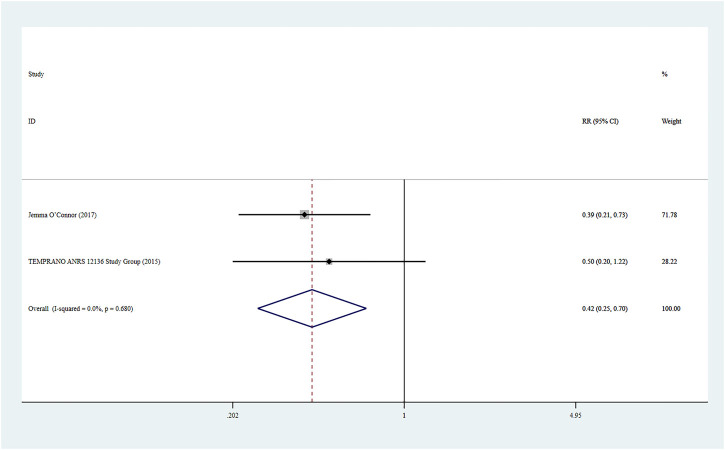
Forest plot for Meta-analysis of severe bacterial infections.

Two studies (O’Connor et al., 2017) reported severe bacterial infections, and a fixed-effect model was used for the combined effect size (*I* ^*2*^ < 0.01%, *p* = 0.680). The combined results indicated a statistically significant difference between the two groups (RR = 0.42, 95% CI: 0.25–0.70, Z = 3.33, *p* = 0.001), suggesting that Rapid ART had a lower risk of severe bacterial infections than standard/delayed treatment. See Figure 8.

## Discussion

Risk of infected tuberculosis caused by Rapid ART standard/delayed treatment. A total of 10 articles were included in this meta-analysis, nine of which were randomized controlled trials, and they were assessed as high-quality literature by the Cochrane Collaboration Risk of Bias Tool. The meta-analysis results showed that compared with standard/delayed treatment, Rapid ART was statistically significant in the risk of continuing care, tuberculosis and severe bacterial infections, and could significantly reduce the risk of tuberculosis and severe bacterial infections (RR = 1.13, 95% CI: 1.03–1.25, *Z* = 2.44, *p* = 0.015) (RR = 0.41, 95% CI: 0.27–0.62, *Z* = 4.24, *p* < 0.001) (RR = 0.42, 95% CI: 0.25–0.70, *Z* = 3.33, *p* = 0.001). There was no statistical difference in adverse events (RR = 0.52, 95% CI: 0.16–1.76, *Z* = 1.05, *p* = 0.294), mortality (RR = 0.77, 95%CI: 0.47–1.24, *Z* = 1.09, *p* = 0.277), and viral load <100 copies/mL at 12 months after treatment (RR = 1.05, 95% CI: 0.80–1.39, *Z* = 0.35, *p* = 0.726). The results indicate that Rapid ART has clinical significance in ensuring the drug safety of patients and reducing the risk of HIV-related diseases in patients.

Currently, the benefits and risks of Rapid ART are still controversial. Mateo-urdiale (Mateo-Urdiales et al., 2019) found that Rapid ART can reduce HIV-related morbidity and mortality and inhibit HIV viral load based on several randomized trials and observational studies. Ford (Ford et al., 2018) found in a randomized trial that Rapid ART shortened the virus inhibition time, but the mortality rate was not significantly reduced. And Rapid ART significantly increased the treatment cost, and loss to follow-up in observational studies showed an increasing trend. According to literature reports, most of the included literature in the current meta-analyses related to Rapid ART were observational studies in Africa. Although the feasibility of Rapid ART has been confirmed, it is difficult to measure the impact on the overall HIV epidemic. This study is a meta-analysis based on multi-region and multi-center RCTs, which confirmed that compared with standard/delayed treatment, Rapid ART was statistically significant in continuing care, and could reduce the risk of tuberculosis and severe bacterial infections in HIV patients.

In addition, our findings revealed that the two treatment choices had a similar risk of adverse events, viral load <100 copies/mL at 12 months following treatment, and mortality, which differed from previous findings. In comparison with standard/delayed treatment, a cohort observational study showed that Rapid ART reduced the possibility of virus inhibition at 6 months (Ford et al., 2018), and a study of adults in South Africa reported a high virus inhibition rate of 94% among HIV-infected people (Wilkinson et al., 2015). This may be due to the different definitions of virus inhibition and Rapid ART in our included literature. Mateo-urdiales (Mateo-Urdiales et al., 2019) included seven studies and found no statistical difference between the two treatments, which is consistent with the results of this study. The possible reason is that the longest follow-up time in the included literature is 3 years, and AIDS has become a chronic disease under the control of ART, so the follow-up time is not enough to draw a conclusion. Adverse events were not analyzed due to the outcome measures of the included literature (Ford et al., 2018; Mateo-Urdiales et al., 2019) as only two trials included in this study used adverse events as an outcome measure, thereby lacking sufficient evidence to draw conclusions on the occurrence of adverse events.

This meta-analysis provides the following benefits. First, this meta-analysis provided a complete and systematic evaluation of the advantages and disadvantages of rapid initiation of antiretroviral therapy against standard/delayed therapy, offering a reference for the selection of following clinical treatment options based on high-quality research. Second, compared with trials that only covered the African region, this study included five well-designed multi-center and multi-region RCTs with a total of 21,543 participants, including multiple ethnic groups and non-short-term treatment outcomes.

However, there are some limitations in this study. First, after thoroughly searching major databases, there were just a few publications that could be included in our meta-analysis. Second, the longest follow-up time of the included literature is 3 years, while AIDS has become a chronic disease under the control of ART. More outcome measures need to be confirmed by subsequent studies with longer follow-up times.

## Conclusion

Rapid ART can reduce the risk of tuberculosis and severe bacterial infections in HIV patients compared to standard/delayed treatment and appears to have a similar safety profile. These findings provide support for the recommendation of WHO to accelerate the initiation of antiretroviral therapy, and Rapid ART is suggested to be used when medical conditions and the patient’s physical conditions permit. However, this meta-analysis included few studies and failed to effectively evaluate treatment-related adverse events, mortality, and virus inhibition. Therefore, we hope that there will be unified standards for the implementation time and virus inhibition of Rapid ART in the future, and more reasonably designed multi-center RCTs. More attention should be paid to the survival and quality of life of patients, and more reports with continuous follow-up need to be made on the outcomes of Rapid ART such as adverse events, to provide sufficient evidence for the treatment of Rapid ART.

## Data Availability

The original contributions presented in the study are included in the article/Supplementary Material, further inquiries can be directed to the corresponding authors.
